# A Case of Tuberculosis-related Cerebral Venous Sinus Thrombosis and Pulmonary Thromboembolism Successfully Treated with Edoxaban

**DOI:** 10.1016/j.rmcr.2022.101736

**Published:** 2022-09-10

**Authors:** Koichi Nishino, Takashi Akimoto, Hideyuki Mitsuoka, Yutaka Terajima, Yuta Arai, Yoshihiro Masui, Tomoyasu Mimori, Kengo Koike, Kazuhisa Takahashi, Mitsuaki Sekiya

**Affiliations:** a Department of Respiratory Medicine, Saiseikai Kawaguchi General Hospital, Japan; b Division of Respiratory Medicine, Juntendo University Faculty of Medicine and Graduate School of Medicine, Japan; c Department of Neurosurgery, Saiseikai Kawaguchi General Hospital, Japan; d Department of Cardiology, Saiseikai Kazo Hospital, Japan

**Keywords:** Cerebral venous sinus thrombosis, Pulmonary thromboembolism, Pulmonary tuberculosis, Venous thrombosis, Direct oral anticoagulant, Edoxaban

## Abstract

A 22-year-old woman was admitted to the hospital with complaints of headache and vomiting. Radiological examinations revealed cerebral sinus venous thromboses, pulmonary thromboembolism, and cavities in the left upper lung. Pulmonary tuberculosis was diagnosed based on sputum and gastric aspirate culture. Heparin followed by warfarin was administered. Anti-tuberculosis agents including rifampicin were also initiated. Since the effect of warfarin did not reach the therapeutic level because of interaction with rifampicin, edoxaban was administered and thromboses were ameliorated. This report illustrates rare thrombotic complications in a TB-induced hypercoagulable state and the potential benefits and safety of edoxaban in combination with rifampicin.

## 1 Introduction

Tuberculosis (TB) can lead to various complications during the acute phase of infection. Pulmonary thromboembolism (PTE) and deep venous thrombosis (DVT) are associated with active pulmonary TB [1]. Cerebral venous sinus thrombosis (CVST) has also been reported in TB patients [2]. These are relatively uncommon but potentially lethal complications. Additionally, treatment for thrombosis can be challenging because of interactions between anticoagulants and rifampicin [3]. This report describes the case of a young woman who developed acute PTE and CVST as complications with pulmonary TB and was successfully treated with anti-tuberculosis agents and edoxaban, a direct oral anticoagulant (DOAC).

## 2 Case report

The patient was a 22-year-old Asian woman who presented with headache, nausea, and vomiting, which had started one day before admission. Fever, dyspnea, chest pain, and cough were absent. She denied the use of oral contraceptives, corticosteroids, hormonal replacement therapy, erythropoietin, or antipsychotic drugs. She also had no history of thromboembolism, immobilization, smoking, pregnancy, abortion, or any significant family history, including thromboembolism.

On physical examination, her body mass index was 18.4 kg/m^2^, and vital signs were within normal limits. Her lung sounds were clear and cardiovascular and abdominal examinations were unremarkable. Her legs were unswollen and Homan's sign was negative. She was alert and oriented, and no muscle weakness or sensory disturbances were found. Photophobia, neck stiffness, and Kernig's sign were absent.

Laboratory findings on admission are shown in Table 1. Briefly, the blood test revealed normal white blood cell and platelet count, elevated C-reactive protein, D-dimer, and fibrin degradation product. Activation of antithrombin III (AT-III) and proteins S and C were within normal limits. Lupus anticoagulant, anti-nuclear and anti-cardiolipin antibodies were also negative. The interferon-γ releasing assay (IGRA) was positive, however, initial acid-fast staining of sputum and gastric aspirate, and polymerase chain reaction for *Mycobacterium tuberculosis* were negative.

**Table 1:** Laboratory data on admission.

Hematology			Immunology			Coagulation		
WBC	5600	g/dL	C3	156	mg/dL	PT-INR	1.17	
Hemoglobin	12.0	g/dL	C4	51	mg/dL	APTT	36	sec
Platelet	16.1	10^4^ μL	IgG	1437	mg/dL	D-dimer	15.4	μg/mL
			IgA	247	mg/dL	FDP	39.2	μg/mL
**Biochemistry**			IgM	106	mg/dL	Fibrinogen	253	mg/dL
Total protein	6.6	g/dL	Anti-nuclear antibody	<40		TAT complex	6.8	ng/mL
Albumin	3.2	g/dL	Anti-cardiolipin antibody	<8.0	U/mL	PAI-1	31	ng/mL
BUN	3.8	mg/dL				SFMC	25.4	μg/mL
Creatinine	0.42	mg/dL	Anti-DNA antibody	<2.0	IUmL	Antithrombin-III activity	82	%
AST	12	IU/L	PR3-ANCA	<1.0	U/mL	Protein C activity	91	%
ALT	9.0	IU/L	MPO-ANCA	<1.0	U/mL	Protein S activity	67	%
LDH	198	IU/L	IGRA (T-SPOT)	**+**		Lupus anticoagulant	−	
ALP	61	IU/L	Anti-MAC antibody	−				
γGTP	12	IU/L	β-D-glucan	<5.0	pg/mL	**Analysis of acid-fast bacilli**		
Na	138	mmol/L	Aspergillus antigen	−		Sputum		
K	3.3	mmol/L	Cryptococcus antigen	−		smear/culture/TB-PCR	**−/+/−**	
Cl	101	mmol/L				Gastric aspirate		
HbA1c	6.0	%				smear/culture/TB-PCR	**−/+/−**	
CRP	3.1	mg/dL						

Abbreviations: WBC, white blood cell; BUN, blood urea nitrogen; AST, aspartate aminotransferase; ALT, alanine aminotransferase; LDH, lactate dehydrogenase; ALP, Alkaline phosphatase; γ-GTP, γ-glutamyl transpeptidase; CRP, C-reactive protein; MPO-ANCA, myeloperoxidase-anti-neutrophil cytoplasmic antibody; PR3-ANCA, proteinase 3-anti-neutrophil cytoplasmic antibody; IGRA, interferon-gamma release assay; MAC, Mycobacterium avium complex; PT-INR, prothrombin time-international normalized ratio; APTT, activated partial thromboplastin time; FDP, fibrin degradation products; TAT, thrombin-antithrombin; PAI-1, Plasminogen activator inhibitor type 1; SFMC, soluble fibrin monomer complex; TB, tuberculosis; PCR, polymerase chain reaction.

**Fig. 1:**
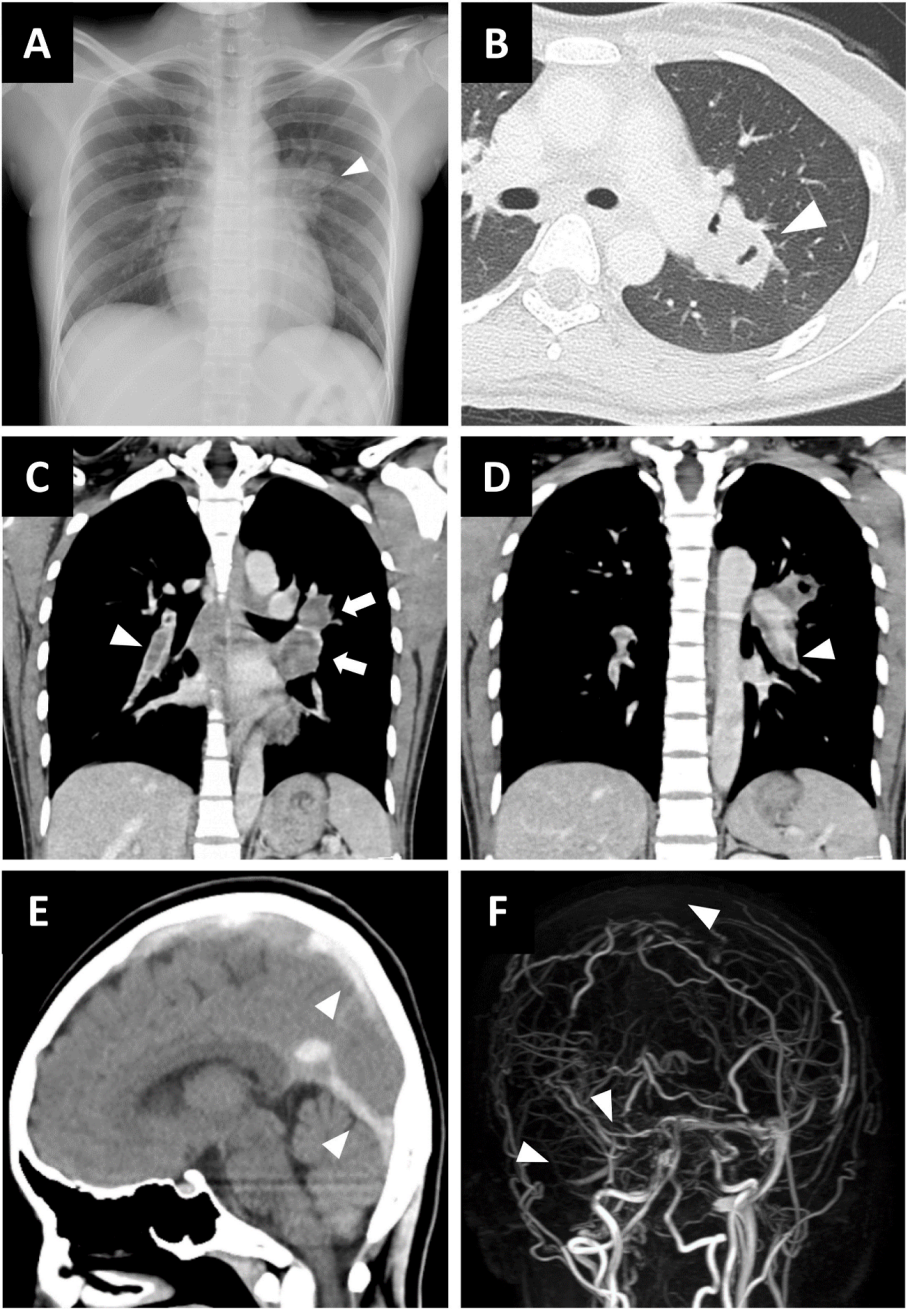
Radiological findings on admission A. Chest radiography showing a left hilar protruding shadow (arrowhead). B. Chest computed tomography (CT) indicating thick-walled lesions with cavities in the left upper lung (arrowhead). C-D. Contrast-enhanced CT of the chest revealing thrombosis in bilateral pulmonary arteries (arrowheads) and left hilar lymphadenopathy (arrows). E. Head CT (sagittal view) demonstrating high-density lesions in the superior sagittal and straight sinus (arrowheads). F. Magnetic resonance venography (MRV) of the brain. Loss of venous signals is seen in the superior sagittal, transverse, and sigmoid sinus (arrows), suggestive of acute thrombosis.

Chest radiography revealed a protruding shadow in the left hilum (Fig. 1A). Chest computed tomography (CT) revealed thick-walled cavitating lesions in the upper left lung (Fig. 1B). Contrast-enhanced CT revealed thromboses in the bilateral pulmonary arteries and lymphadenopathy in the left hilum (Fig. 1C–D). No mass suspicious of cancer was found in a whole-body contrast-enhanced CT. DVT in the lower limbs was not detectable on contrast-enhanced CT or Doppler ultrasonography. CT of the head revealed high-density lesions in the superior sagittal, straight, and sigmoid sinuses (Fig. 1E). Magnetic resonance venography (MRV) showed loss of signals in the superior sagittal, transverse, and sigmoid sinuses (Fig. 1F).

**Fig. 2:**
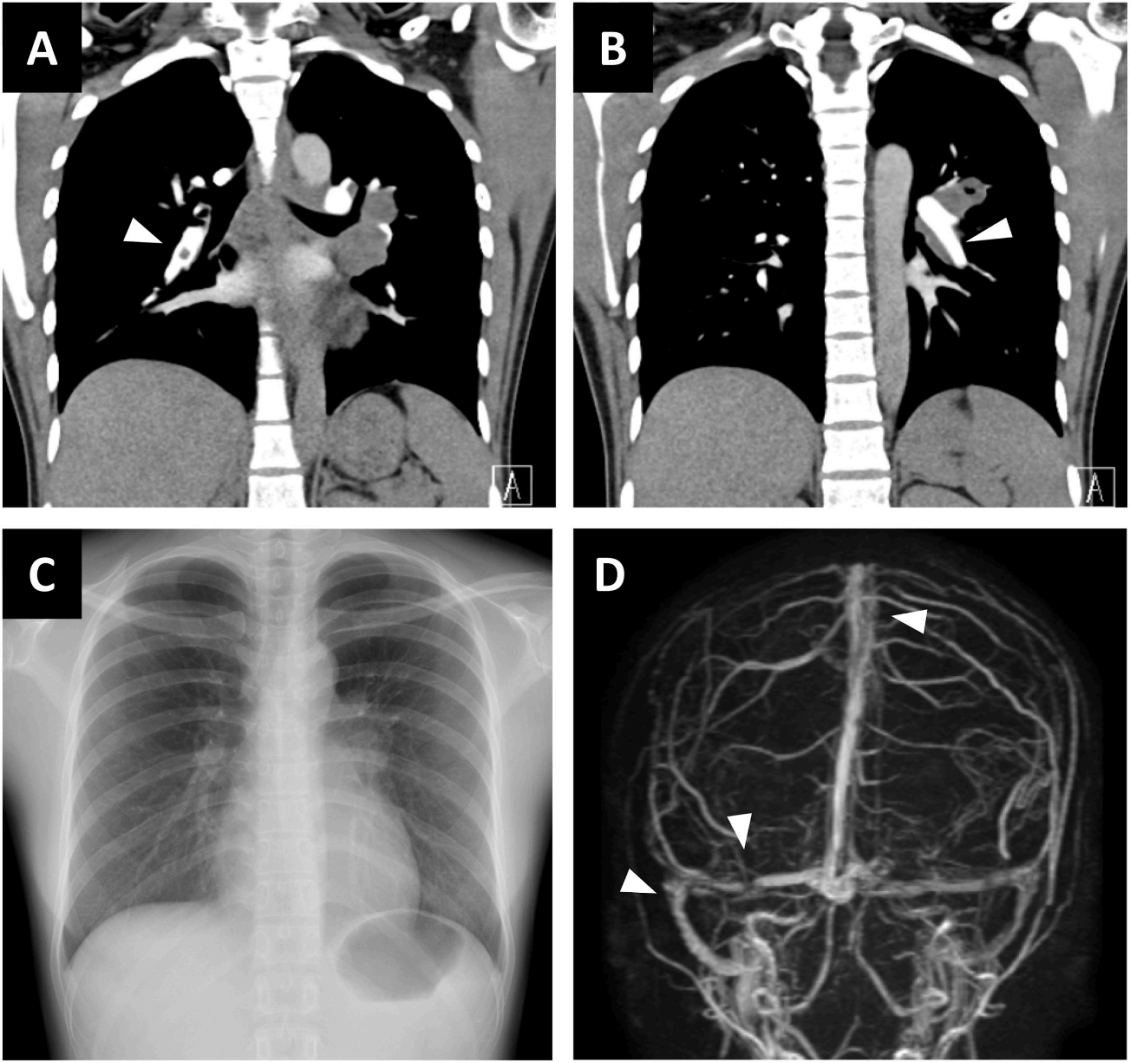
Radiological findings at follow-up A-B. Contrast-enhanced CT of the chest three weeks after the admission. Note that marked amelioration of thromboses in bilateral pulmonary arteries (arrowheads). C. Chest radiography after completion of anti-tuberculous therapy showing shrinkage of the left hilar lymphadenopathy. D. MRV after completion of edoxaban therapy indicating recovery of the venous blood flow in the right cerebral venous sinuses (arrows).

Thus, a diagnosis of acute PTE and CVST was confirmed and intravenous heparin (15000 U/day) was administered. We also initiated glycerol and levetiracetam (200 mg/day) because she developed right-sided hemiplegia and mildly impaired consciousness caused by brain edema and non-convulsive epilepsy after admission. Although TB was not confirmed on admission, active pulmonary TB was strongly suspected based on the radiological findings and the positive result of the IGRA test. Accordingly, isoniazid (220 mg/day), rifampin (450 mg/day), ethambutol (750 mg/day), and pyrazinamide (1100 mg/day) were initiated. After two weeks of heparin administration, we switched to warfarin (3 mg/day). However, the prothrombin time-international normalized ratio (PT-INR) did not reach the therapeutic range even with an increased dose of warfarin (5 mg/day) because of the inhibitory effect of interaction with rifampicin. Therefore, treatment with edoxaban (30 mg/day) was initiated. After three weeks of admission, a followed-up CT scan revealed the amelioration of the cerebral sinus thrombosis (not shown) and bilateral pulmonary thromboembolism (Fig. 2A–B). Her symptoms completely resolved and she was discharged from our hospital. *M. tuberculosis* was confirmed in both sputum and gastric aspirates after eight weeks of culture. She continued standard anti-TB therapy and anticoagulation therapy for six months. After the completion of the treatment, a follow-up chest radiography showed shrinkage of the left hilar lymphadenopathy, and MRV of the brain indicated recovery of the venous blood flow in the cerebral sinuses (Fig. 2C–D).

## 3 Discussion

We herein report a rare case of pulmonary TB along with development of multiple venous thromboses. To the best of our knowledge, our case is the first report of a patient who was successfully treated with edoxaban in combination with rifampicin for tuberculosis-related CVST and PTE.

Venous thrombosis can occur as a complication in TB. A systematic review reported that the prevalence of PTE and DVT was 3.5% in patients with active TB, which is higher than that in the normal population [4,5]. In contrast, CVST is a rare complication of TB, with only a few cases reported [2]. Accordingly, we believe that ours is an extremely rare case where PTE and CVST developed simultaneously.

A hypercoagulable state, endothelial damage, and venous stasis may play a role in the occurrence of thrombosis. Primary hypercoagulable states are caused by hereditary disorders including antiphospholipid syndrome, antithrombin III deficiency, factor V Leiden, prothrombin mutation, lupus anticoagulant, and protein C or S deficiency. In our patient, despite the young age and development of multiple thromboses, these hereditary coagulation disorders were unlikely based on her Asian race, the absence of abnormalities in anticoagulation factors or any personal or family history of thrombosis. Tuberculosis can lead to a secondary hypercoagulable state. Previous studies have observed activated coagulation (elevated plasma fibrinogen, factor VIII, and thrombin-antithrombin complex) and inhibition of fibrinolysis (decreased plasma AT-III and protein C and S) in patients with pulmonary TB [6,7]. These hematological changes probably result from the production of interleukin-6 and tumor necrosis factor-alpha triggered by *M. tuberculosis* infection [8]. TB also induces activation of vascular endothelial cells which can promote coagulation [9]. Although venous stasis can occur via compression of veins by TB lesions [10], venous compressions were not detectable by radiological examinations in our patient. The risk factors of venous thrombosis such as obesity, old age, malignancy, major surgery, oral contraceptives, and a bedridden state are well-known [11]. However, these were absent in our patient based on her medical history and physical examinations. CVST can be caused by various disorders of hypercoagulable states, head trauma, intracranial procedures, brain tumors, abscess, and bacterial meningitis [12]. Since most CVST cases with TB occur as a complication of tuberculous meningitis [2,13,14], it may be possible that our patient suffered from tuberculosis meningitis. However, we could not perform a lumbar puncture for cerebrospinal fluid analysis because of the urgent need for anticoagulation therapy. Among the differential diagnosis which can trigger venous thrombosis, we considered the fact that our patient developed acute PTE and CVST mainly due to a secondary hypercoagulable state caused by active pulmonary TB.

Current guidelines recommend the use of DOACs for acute PTE rather than warfarin after heparin administration [15] On the other hand, warfarin is a standard treatment for CVST, and DOACs are not recommended because of limited evidence [16]. The use of oral anticoagulants for patients with TB is often a major concern for clinicians. Rifampicin, a key drug for TB, decreases the effect of anticoagulants by inducing the activity of various drug transporting and metabolizing enzymes [3]. Clinicians frequently experience difficulties in achieving therapeutic PT-INR levels, even with significant increases in warfarin doses [17]. The use of DOACs for patients with TB-associated thrombosis is expected to increase because of their efficacy and safety [18]. However, DOACs should also be used with caution because rifampicin reduces the area under the curve (AUC) of DOACs. We decided to use edoxaban because the degree of AUC decrease of edoxaban (34%) by rifampicin could be smaller than that of apixaban (53%), dabigatran (67%), and rivaroxaban (49%) [3]. Indeed, edoxaban showed sufficient thrombolytic effect in our patient. Rifabutin, an alternative of rifamycin, seems to have fewer interactions with edoxaban; however, sufficient pharmacokinetic data are still lacking [3]. Further clinical trials are required to evaluate the safety and efficacy of DOACs in the management of TB-associated thrombosis.

## 4 Conclusion

We experienced a rare case of pulmonary TB involving the development of acute PTE and CVST. Our case illustrates the importance of recognizing the possibility of a hypercoagulable state and careful evaluation for systemic thromboses in a patient with active TB. Although drug interactions between oral anticoagulants and rifampicin are often problematic, this report suggests that treatment with edoxaban in combination with rifampicin is potentially beneficial and safe.

## Funding sources

This research did not receive any specific grant from funding agencies in the public, commercial, or not-for-profit sectors.

## Ethics approval and consent to participate

This report was approved by the Ethical Committee of Saiseikai Kawaguchi General Hospital. Written informed consent was obtained from the patient for publication of this case report.

## Declaration of competing interest

We have had no prior discussions with a Respiratory Medicine Case Reports Editorial Board Member about the work described in our manuscript. The manuscript has never been published and is not under consideration for publication elsewhere. All the authors have read the manuscript and approved this submission. The authors have no conflicts of interest to declare.
